# Physical Activity Complexity and Cognitive Function in Older Adults

**DOI:** 10.1093/geroni/igaf122.267

**Published:** 2025-12-31

**Authors:** Yurun Cai, Beth Snitz, Ann Cohen

**Affiliations:** University of Pittsburgh, Pittsburgh, Pennsylvania, United States; University of Pittsburgh, Pittsburgh, Pennsylvania, United States; University of Pittsburgh, Pittsburgh, Pennsylvania, United States

## Abstract

Physical activity (PA) is associated with cognitive impairment and dementia in older adults. However, most studies have focused solely on quantities or intensities (e.g., moderate to vigorous PA) which may introduce measurement error. This study aims to examine the association between a novel PA measure–PA complexity, derived from continuous accelerometer signals using multiscale entropy method–and cognitive function in older adults in the Connectomics in Brain Aging and Dementia study. Among 190 adults who completed neuropsychological tests and 7-day wrist-worn accelerometer assessment in 2016-2021, 166 (mean age=65.3±8.9y) had ≥3 valid days (≤10% non-wear time of the day) of accelerometer data and were included in the analysis. Confirmatory factor analysis was used to construct three factors (memory, language, and executive function) from neuropsychological tests. In the unadjusted piecewise linear regression models, every 0.1-unit higher in PA complexity was associated with a 3.2-unit (p = 0.004) higher fluid cognition composite score and a 2.7-unit (p = 0.008) higher total cognition composite score when PA complexity was ≤0.6. After adjusting for sociodemographic characteristics and total activity counts per day, every 0.1-unit higher in PA complexity was associated with a 1.8-unit (p = 0.044) higher total cognition composite score. Low PA complexity (≤0.6) was associated with lower factor scores in memory (β=-0.26, p = 0.037), language (β=-0.33, p = 0.008), and executive function (β=-0.37, p = 0.004). Our findings highlight the importance of assessing novel PA metrics which may be more sensitive than conventional metrics to identify subtle changes in activity patterns and help predict early stages of pathological progression to dementia.

